# Immunotherapeutic implications of negative regulation by protein tyrosine phosphatases in T cells: the emerging cases of PTP1B and TCPTP

**DOI:** 10.3389/fmed.2024.1364778

**Published:** 2024-04-19

**Authors:** Luis Alberto Perez-Quintero, Belma Melda Abidin, Michel L. Tremblay

**Affiliations:** ^1^Rosalind and Morris Goodman Cancer Institute, Faculty of Medicine, McGill University, Montreal, QC, Canada; ^2^Department of Biochemistry, McGill University, Montreal, QC, Canada

**Keywords:** protein tyrosine phosphatases, T cells, immunotherapy, signaling, PTP1B (PTPN1), TCPTP (PTPN2)

## Abstract

In the context of inflammation, T cell activation occurs by the concerted signals of the T cell receptor (TCR), co-stimulatory receptors ligation, and a pro-inflammatory cytokine microenvironment. Fine-tuning these signals is crucial to maintain T cell homeostasis and prevent self-reactivity while offering protection against infectious diseases and cancer. Recent developments in understanding the complex crosstalk between the molecular events controlling T cell activation and the balancing regulatory cues offer novel approaches for the development of T cell-based immunotherapies. Among the complex regulatory processes, the balance between protein tyrosine kinases (PTK) and the protein tyrosine phosphatases (PTPs) controls the transcriptional and metabolic programs that determine T cell function, fate decision, and activation. In those, PTPs are *de facto* regulators of signaling in T cells acting for the most part as negative regulators of the canonical TCR pathway, costimulatory molecules such as CD28, and cytokine signaling. In this review, we examine the function of two close PTP homologs, PTP1B (PTPN1) and T-cell PTP (TCPTP; PTPN2), which have been recently identified as promising candidates for novel T-cell immunotherapeutic approaches. Herein, we focus on recent studies that examine the known contributions of these PTPs to T-cell development, homeostasis, and T-cell-mediated immunity. Additionally, we describe the signaling networks that underscored the ability of TCPTP and PTP1B, either individually and notably in combination, to attenuate TCR and JAK/STAT signals affecting T cell responses. Thus, we anticipate that uncovering the role of these two PTPs in T-cell biology may lead to new treatment strategies in the field of cancer immunotherapy. This review concludes by exploring the impacts and risks that pharmacological inhibition of these PTP enzymes offers as a therapeutic approach in T-cell-based immunotherapies.

## Introduction

Recent developments in cancer immunotherapy have identified PTP1B as a potential pharmacological target to enhance anti-tumoral responses (1–4). PTP1B holds a significant position as a regulator of Janus kinase/signal transducer and activator of transcription (JAK/STAT) and Receptor Tyrosine Kinases (RTKs). In conjunction with TCPTP, a homologous and more extensively studied partner of PTP1B, the inhibition of both phosphatases has proven to be a highly specific and potent strategy for the reactivation of immune cellular responses, offering an alternative to checkpoint blockade (5). In this review, we will specifically address the known roles of both enzymes in T cells and the potential mechanisms of action in these cells for the novel class of PTP1B/TCPTP inhibitors.

The ability of T cells to mount robust, antigen-specific responses places them among the central players of adaptive immunity (6, 7). However, T-cell activation is stringently regulated to promote pathogen clearance while maintaining tolerance. Signaling networks, including protein tyrosine kinases (PTKs) and PTPs, actively participate in the balance between T-cell activation and inhibition, determining the extent of T-cell activation and effector functions (8).

T cell activation is triggered by the integration of three major signals: (1) antigen recognition by T cell receptor (TCR), (2) costimulatory signals, and (3) cytokines and their receptors (9). TCR activation is induced upon stimulation by peptide-bound class I or class II major histocompatibility complexes (pMHCI or pMHCII) on the surface of antigen-presenting cells (APCs). Activating signal transduction from the TCR is exclusively mediated by the tyrosine kinases lymphocyte-specific protein tyrosine kinase (Lck), Fyn, and zeta-chain-associated protein 70 (ZAP-70) which sequentially phosphorylate and recruit downstream effectors such as the linker for activation of T cells (LAT), SH2-domain-containing leukocyte protein of 76 kDa (SLP76), Phospholipase C gamma (PLC-γ), Vav, growth factor receptor bound protein 2 (Grb2)-son of sevenless (SOS) and extracellular signal-regulated kinases (ERK). These processes have been reviewed in more detail elsewhere (9–11).

Without costimulatory signals, T cells would become anergic and refractory to future stimulation (12). Hence, at this point, costimulatory receptors such as CD28 and inducible T cell costimulatory (ICOS) are simultaneously engaged to ensure signal amplification of TCR-initiated pathways, such as phosphatidylinositol 3-kinases (PI3K), interleukin-2-inducible T-cell kinase (Itk)-PLC-γ and Grb2-VAV, allowing continuation toward T cell activation. The ligands that bind to these coreceptors include B7.1/B7.2 and ICOS ligand (ICOSL) respectively. A more detailed review of costimulation in T cell activation can be found elsewhere (9–11).

While this complex signaling network ensures a specific and selective response, cytokine cues serve as a third signal, to further regulate the epigenetic landscape and transcriptional programs in activated T cells (11, 13, 14). Cytokine signals are primarily transduced by tyrosine phosphorylation driven by the JAK/STAT signaling network, composed of four members of the JAK non-receptor tyrosine kinases and seven members of the phosphorylated STAT transduction factors. Since the early characterization of T helper cell (Th) responses, specific cytokines have been associated with different types of immune responses. For example, the presence of interferons was related to the activation of cytotoxic mechanisms important in viral control, albeit the presence of IL-4 was associated with potent antibody responses (15). As such, it has been found that after activation, naïve CD4 T cells differentiate into functional subsets of T helper (Th) cells including but not limited to Th1, Th2, Th17, and Treg cells in response to different cytokine combinations. For instance, Th1 requires a combination of interleukin 12 (IL-12) and interferon-gamma (IFN-γ); Th2 develops in response to IL-4 and IL-2, and Th17 is commanded by transforming growth factor beta (TGFβ) and IL-6, IL-21 or IL-23 (13, 16, 17). Aberrant activation of Th subsets is the hallmark of organ-specific autoimmune diseases and failed antitumoral responses.

Activation of T cell responses is the last resource in the arsenal available to the immune system. It is engaged when barrier and local innate mechanisms have failed. *Per se*, it exerts extensive pressure on the body’s economy and exposes it to harmful off-target effects by inflammatory mediators. To maintain health, regulation, and termination of the immune responses are as required as activation itself (5). As tyrosine phosphorylation plays a crucial role in the intracellular transduction process involved in T cell activation PTPs and the pathways that recruit them act as essential regulators in this process. At the costimulatory level, regulators of these interactions, include the expression of inhibitory checkpoint receptors such as programmed cell death protein 1 (PD1) and Cytotoxic T-lymphocyte associated protein 4 (CTLA-4) which are rapidly induced following activation helping to prevent unwanted damage (11, 13, 14). To this end, PD1 and CTLA4 recruit the PTPs Src homology region 2 domain-containing phosphatase1 (SHP1) and SHP2 to the activation site, known as the immunological synapse (18–20). However, the mechanisms of these pathways differ. PD1 responds to the engagement of PD1 ligands 1 and (PDL1/2), which are present on the cell targets and APCs, by directly recruiting SHP2. On the other hand, CTLA-4 functions by competing with CD28 ligands B7.1/B7.2 (21). SHP1 and SHP2 activity is observed upon CTLA engagement, it is proposed to be the result of indirect interactions (22). Targeted blockade of these signals results in the reactivation of tumor immunity, which is currently the most successful form of immunotherapy for cancer (23).

Likewise, tyrosine phosphatases play an exquisite role in the regulation of the JAK/STAT pathway. Inhibition of these pathways has also emerged as a promising target for pharmacological intervention (5, 24). This significant role makes PTP1B and TCPTP noteworthy candidates for the development of cancer-targeting immunotherapies.

## Overview of PTP1B and TCPTP

PTP1B and TCPTP are two closely related class 1 protein tyrosine phosphatases (PTP) (25). They both belong to the non-receptor tyrosine phosphatases (NRPTP) family and share 74% identity in their catalytic domains (26). They are found to be ubiquitously expressed (27, 28) though their expression is tightly regulated, such is the case for PTP1B during metabolic homeostasis (29), and cell cycle or thymocyte development for TCPTP (30, 31). TCPTP was one of the first identified NRPTP, it was cloned from a T cell cDNA library, hence its name (32). Its gene produces several messenger RNAs by alternative splicing. Among those, the two main messengers differ on their 3′ last coding exons which give rise to a shorter 45kD protein found primarily in the nucleus, that can translocate to the cytoplasm under certain stimuli. The longest main mRNA encodes for a 48kD protein form that locates to the cytoplasm via a hydrophobic motif (30, 33, 34).

PTP1B and TCPTP are well-established regulators of several members of the JAK/STAT signaling network, acting at different levels from the cell membrane to the nucleus. They target the activating trans-phosphorylated residues of JAKs and the C-terminal dimerizing tyrosine residues of STATs, limiting downstream phosphorylation and preventing dimerization and nuclear translocation of STATs (24). Due to the essential role of JAK and STAT proteins in cytokine signal transduction, these phosphatases, particularly TCPTP, have received significant attention in their immunological roles. Early studies on TCPTP’s immunoregulatory role date back to the generation of germline mouse deletion models, making it the first genetic knockout mouse line for any PTP. These models exhibited a severe phenotype characterized by immune dysregulation, leading to premature death (35, 36). Homozygous mutants were viable after birth but died 3–4 weeks of age, displaying prominent signs of inflammation characterized by mononuclear infiltration in non-lymphoid organs and the presence of high levels of pro-inflammatory factors such as IFN-γ, tumor necrosis factor alpha (TNF-α) and the inducible form of nitric oxide synthase (iNOS). Interestingly, the source of these cytokines was found to be non-hematopoietic in origin, specifically originating from stromal cells in the bone marrow (37). Further studies on these mice also revealed a biased hematopoiesis that limited red blood cell formation and B cell maturation (38).

In contrast, mice deficient in PTP1B were generally healthy and reproduced at Mendelian ratios (39). However, they showed a minor immune system phenotype characterized by the accumulation of B cells in bone marrow and lymph nodes, an alteration that occurred downstream of common lymphoid progenitors (40). Further hematopoietic dysregulation (41, 42) and altered immune reactivity were detected in PTP1B KO mice after specific cytokine challenges (43).

Nevertheless, PTP1B and TCPTP substrates are not exclusively from the JAK/STAT network. Although their regulation of these pathways can account for the strong immunophenotype both enzymes also target several other independent substrates, some of which include potential contributions to T cells that cannot be ignored. This is the case for members of the RTK (44–49), Src kinases (31, 50), phosphorylated adaptors (51–55), viral sensors and transducers (56, 57), and the MAP kinase p38 (58). It is expected that phenotypical differences observed between mice deficient for either of these enzymes arise from their substrate preferences.

## Structure and localization of PTP1B and TCPTP

**Figure 1:**
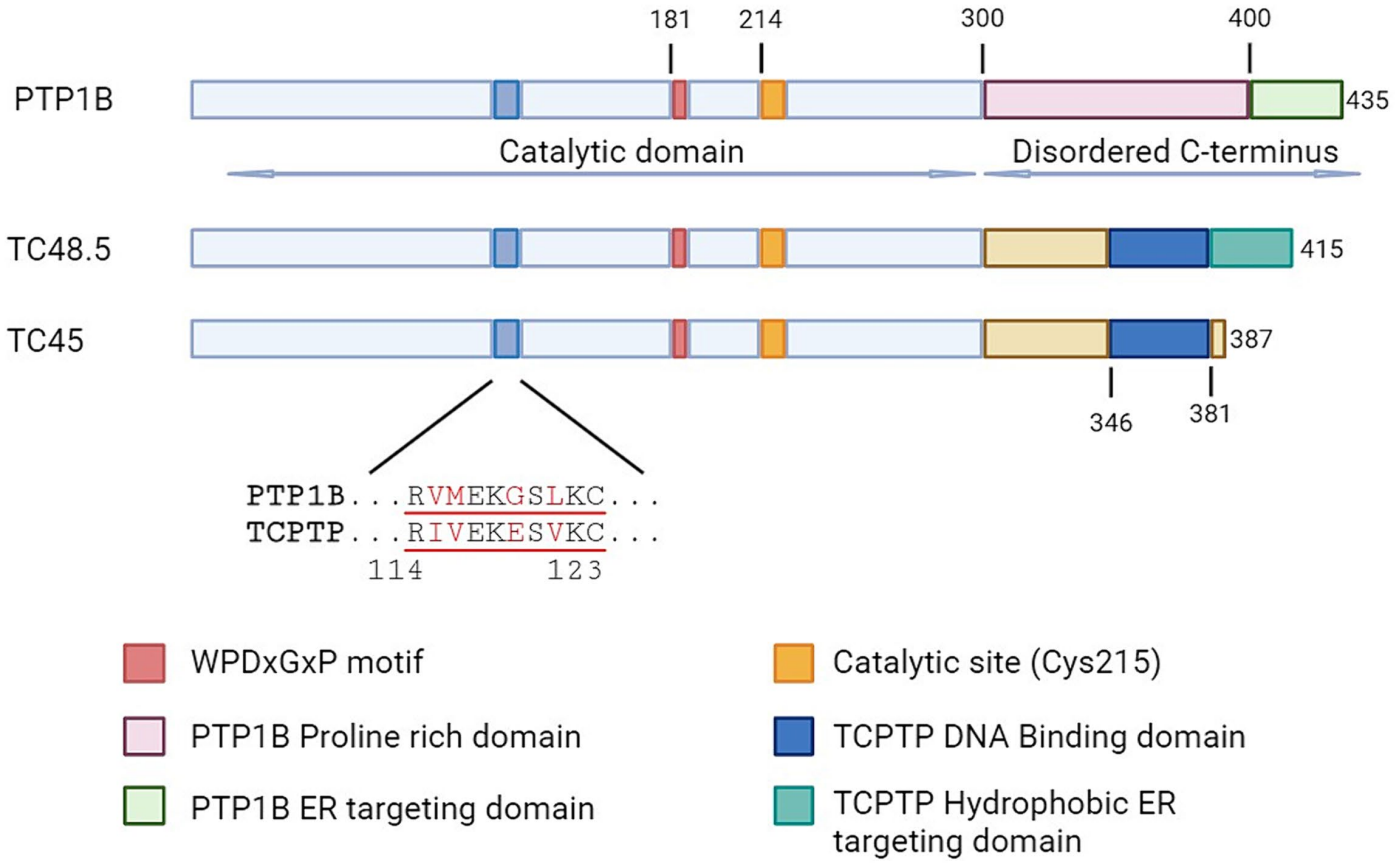
Schematic representation of protein tyrosine phosphatases PTP1B and TCPTP. Aligned representation of the conserved domains, with emphasis on regions involved in functional differences. TC48.5 and TC45 represent the TCPTP isoforms of 48.5 and 45 kDa, respectively.

Both TCPTP and PTP1B have similarities in their catalytic domains, but they differ in their structural features and how they interact with their ligands. These differences contribute to the specific roles of these PTPs in cellular processes. Catalytic domain-swapping experiments have shown that PTP1B and TCPTP exhibit differences in inherent specificity, suggesting that each enzyme has unique substrate preferences (59). While both TCPTP and PTP1B contain certain residues, such as Gly259, variations in other residues contribute to the distinct substrate specificities observed in these two enzymes (59). TCPTP features a less structured domain between Arg114 and Cys123, forming an unstructured loop (60) (Figure 1). This structural characteristic enhances its substrate specificity by facilitating specific amino acid interactions within the active site (60). In the case of PTP1B, Arg47 plays a significant role in determining substrate specificity, leading to a slight preference for acidic residues NH2-terminal to phosphotyrosine (61). This particular amino acid interaction is essential for defining the substrate specificity of PTP1B.

In terms of ligand and protein interactions, there are also variations between the two enzymes. TCPTP interacts with specific amino acids such as Asp181 and Phe182 in the WPD motif, and Cys215, Ala217, and Arg221 in the catalytic site, which influence the specificity of TCPTP (62). On the other hand, PTP1B has unique biophysical characteristics, such as cyclic motions in the WPD loop that regulate the rate of phosphotyrosine hydrolysis (63). Additionally, its activity can be modulated by post-translational modifications outside the active site (63).

Both PTP1B and TCPTP have intrinsically disordered C-terminal (IDR) tails, however lacking the homology observed in the catalytic domain (24) (Figure 1). The function of this tail seems to contribute largely to the intrinsic regulation of the enzymes, nonetheless in an opposite manner. In PTP1B, allosteric ligands bind to an α site between specific helices, affecting the enzyme’s activity and regulatory processes. The binding behavior at this site contributes to the selective inhibitory effects on PTP1B compared to TCPTP (64). Indeed, Trodusquemine (MSI-1436) the first highly selective PTP1B inhibitor acts by binding directly to this site (65). In contrast, the C-terminal tail of the 45 kDa isoform of TCPTP interacts directly with the catalytic site reducing its activity. This autoinhibitory role is achieved not by occupying the catalytic site, but rather by generating a steric bulk that limits the access of the substrate (66). Interactions with the activated form of Integrin α1 (ITGA1) displace TCPTP’s C-terminal domain allowing the full activation of the enzyme.

TC45 is the specific form of TCPTP primarily found in the nucleus of T cells (67) suggesting that in T Cells the activity of TCPTP is controlled by a region at the IDR (68), which can potentially impact phosphorylation of activated STATs (69). On the other hand, the TC48 variant is located in the endoplasmic reticulum (ER) within T cells (67), suggesting that TC48 may be involved in ER-related functions or specific signaling cascades. Yet, the intracellular localization of PTP1B in T cells has not been confirmed. However, research suggests that PTP1B is located within the nucleus in different types of tumor cell lines (70).

## PTP1B and TCPTP in T cells

The strong immune phenotype induced by cells of either hematopoietic or non-hematopoietic origin obscured the observations leading to finding the intrinsic defects that lack of TCPTP may induce in T cells. However, studies of lymphopoiesis with precursors obtained from full-body deficient mice have revealed that TCPTP plays an intrinsic role in the development of common lymphoid progenitors in the bone marrow (37). Additionally, T cell defects that were observed in the initial study of the full-body TCPTP KO mice (35) were further confirmed by studies using conditional deletion driven by the specific expression of CRE under the Lck promoter (31). Moreover, *in vitro* models of thymopoiesis have provided additional insights into the roles of TCPTP in T cell development (71). Interestingly, the conditional deletion of TCPTP in T cells did not affected significantly the lifespan of mice, as observed in full body knockouts, suggesting that T cells only play a minor role in the observed immunopathology (31).

While TCPTP has been extensively studied for its immune roles, its closest related, PTP1B, has only recently been examined for its function in the immune system. PTP1B was the first tyrosine phosphatase to be characterized and purified (72). It was named after the elution peaks where it was identified. PTP1B is still considered the archetypical PTP and its study has helped identify many of the common motifs essential for catalysis, such as the WPD and Cysteine 215 (Figure 1) (73). A decade after its discovery, PTP1B received intense attention thanks to earlier links to blood glucose regulation, as deficient mice were resistant to diet-induced obesity and were insulin hypersensitive (39, 74, 75). However, germline deletion of PTP1B does not have the same dramatic phenotype observed in TCPTP full-body knockout. Instead, PTP1B deficient mice are born at Mendelian ratios from heterozygous parents, gain weight at a similar rate as WT counterparts, are fertile, and have similar life expectancy (39, 75). However, the immune challenge of these mice reveals some phenotypic changes for PTP1B in the myeloid compartment (41, 76). A more globally increased inflammatory response was seen when PTP1B deficient animals were subjected to ovalbumin (OVA) sensitization and further airway challenge (43). Monocyte, neutrophil, eosinophil, and lymphocyte populations were increased in the lungs, along with Th2 type of cytokines. More recently, PTP1B has been proposed as a central mediator in sex-related immune response differences (77), and to have a potential role in regulating TCR signaling (78). In the following sections, we will discuss in-depth the current understanding of the roles that PTP1B and TCPTP have in T cell biology. For a quick reference, please consult Table 1.

**Table 1:** Phenotypic consequences in TCPTP and PTP1B deletion in T cells.

	Observed phenotypes in TCPTP deficient T cells	Ref
T cell development	Favored thymic γδ T cell development Increased ratio of single positive/double positive thymocytes Enhanced IL-7R, IFN-γ and STAT5 signaling in immature thymocytes	(31, 71, 79)
TCR signaling	Dephosphorylation of its direct substrates Lck and Fyn Reduction in the phosphorylation of downstream ERK Enhanced effector phenotype	(31, 79–81)
T cell proliferation	No effect on the homeostatic proliferation of naïve T cells Enhanced lymphopenic T cell expansion	(31, 79–81)
CD4 T cell polarization	Attenuated Th1 differentiation accompanied by enhanced IFNγ-induced STAT1 phosphorylation. Favored Th1 and Th17 polarization following adoptive transfer in TCR transgenic mice and worsened aggressive colitis. Increased disease initiating Th1, Th17 and effector Treg dominant response in a mouse model of arthritis	(71, 82–85)
T cell tolerance	Alterations of the numbers of Treg cells in mice Enhanced stabilization of Foxp3 in *in vitro* induced Treg Destabilization of Foxp3 in effector Tregs in arthritic mice Spontaneous autoreactivity of naïve CD8 T cells	(80, 83, 84, 86)
Cytokine responses	Enhanced IL2 cytokine sensitivity Enhanced IL-7R and IFN-γ signaling	(71, 84, 86–88)
Terminal differentiation	Accumulation of Tim3+ and KLRG1+ CD8 terminal effector T cells	(32, 89)
T cell exhaustion	Despite enhanced TCR and IFN signals, and terminal effector marker expression, CD8 T cells maintain higher proliferative rates	(32, 89)
Tumor immunity	Increased Th1 and Th17 differentiation of CD4 T cells Increased conversion of Tregs to Th17 Enhanced effector functions of CD8 T cells Enhanced proliferation of differentiated effector CD8 T cells Pro-inflammatory and pro-oncolytic effects in other immune cells Increased intrinsic tumor susceptibility to CD8 cells	(1, 32, 35, 83, 85, 90)

	Regulatory role of PTP1B in T cells	Ref
TCR signaling	Inhibits TCR-dependent T cell activation by acting in a feedback loop with adaptor proteins Dok-1 and TRAF3 Its substrate Dok-1 negatively regulates TCR signaling Contributes to the modulation of LAT signalosome during early TCR signal propagation	(51, 78)
T cell polarization	Mediates androgen-induced suppression of Th1 polarization	(53)
Cytokine responses	Dephosphorylates JAK2 and Tyk which play a crucial role in IL12 and IL23 responses	(53)

## PTP1B and TCPTP in T cell development

Development of T cells in the thymus occurs through a well-characterized series of stages (91). The most immature T cell precursors in the thymus are contained in a population that does not express either of the T coreceptors CD4 and CD8, named DN (double negative) thymocytes. These DN cells proceed through four sequential stages (DN1-DN4) in which they rearrange TCRβ, TCRγ, and TCRδ genes subsequently testing their signaling capacity. In case of successful arrangement of the TCRβ chain, cells progress to the next stages in a process known as β selection. They then proceed to a double positive (DP) stage, where immature thymocytes express both CD4 and CD8. At this step, the β chain is paired with the TCR α chain, and thymocytes with low-affinity TCR for self-peptide then differentiate into CD4 or CD8 single positive (SP) thymocytes, according to their MHC preference (92).

Observations from the OP9-DL1 culture system demonstrated that TCPTP negatively regulates IL-7R-induced phosphorylation of STAT1 and STAT5, and its deficiency leads to aberrant IFN-γ signaling in thymocyte progenitors during early T cell development beyond β selection (71, 79). It is worth noting that, IL-7R signaling is not only essential for the survival of TCRβ− DN thymocytes, but has also been implicated in the development and survival of γδ T cells (91). In the thymus, TCPTP protein levels are upregulated from DN1 to DN2 and DN3 then decline at DN4 (80). The transition from DN2 to DN3 stage defines an early T lineage commitment checkpoint before TCR expression (93). Loss of TCPTP has been shown to favor a bias toward γδ T cells at this stage of development, possibly due to enhanced signals from IL-7R, IFN-γ (71), and STAT5 (79). At the DN4 and SP stages, IL-7R signaling is known to be downregulated. Nevertheless, the impact of TCPTP on TCR signals may still be relevant, as optimal activation of the TCR is required for the differentiation into mature SP thymocytes (94). Unlike during the DN development stages, TCPTP protein levels are upregulated as thymocytes transition from DP to the SP stage (79). Before reaching maturity, T cells undergo two other checkpoints known as positive and negative selection, which depend on the quality of the TCR signals. T cell specific-TCPTP deficient mice show enhanced positive selection, as evidenced by increased numbers of SP cells and an increased SP/DP ratio. This promotion of positive selection in TCPTP deficiency was further confirmed in the TCR transgenic OT-I model (81). Upon reaching the periphery, TCPTP deficient T cells exhibit an activated memory phenotype (31) similar to what is observed in mild cases of lymphopenia, potentially due to increased TCR signals during DN3, DN4, and β selection stages. Collectively, the data demonstrate a role for TCPTP during T cell development by limiting the magnitude of signals rising from the TCR and cytokine receptors that, independently or synergistically, contribute to it in a stage-specific manner.

In the periphery, TCPTP also regulates the homeostatic signals of T cells. Under physiological conditions, the major determinant of peripheral naïve T cell turnover is the number of thymic emigrants and the survival rate of these cells in the periphery. While IL7-R mediated signals are required for the survival of naïve T cells, homeostatic proliferation requires incorporated signals derived from both TCR-pMHC and IL7R-IL7 interactions. Although IL-7 signals are capable of promoting the homeostatic proliferation of naïve T cells under lymphopenic conditions (95), TCPTP deficiency in T cells does not affect the homeostatic proliferation of peripheral T cells, however, it does enhance lymphopenic proliferation of naïve T cells through augmented TCR signaling in an IL-7 independent manner (80).

## PTP1B and TCPTP as negative regulators of TCR signaling in naïve CD4 and CD8 T cells

**Figure 2:**
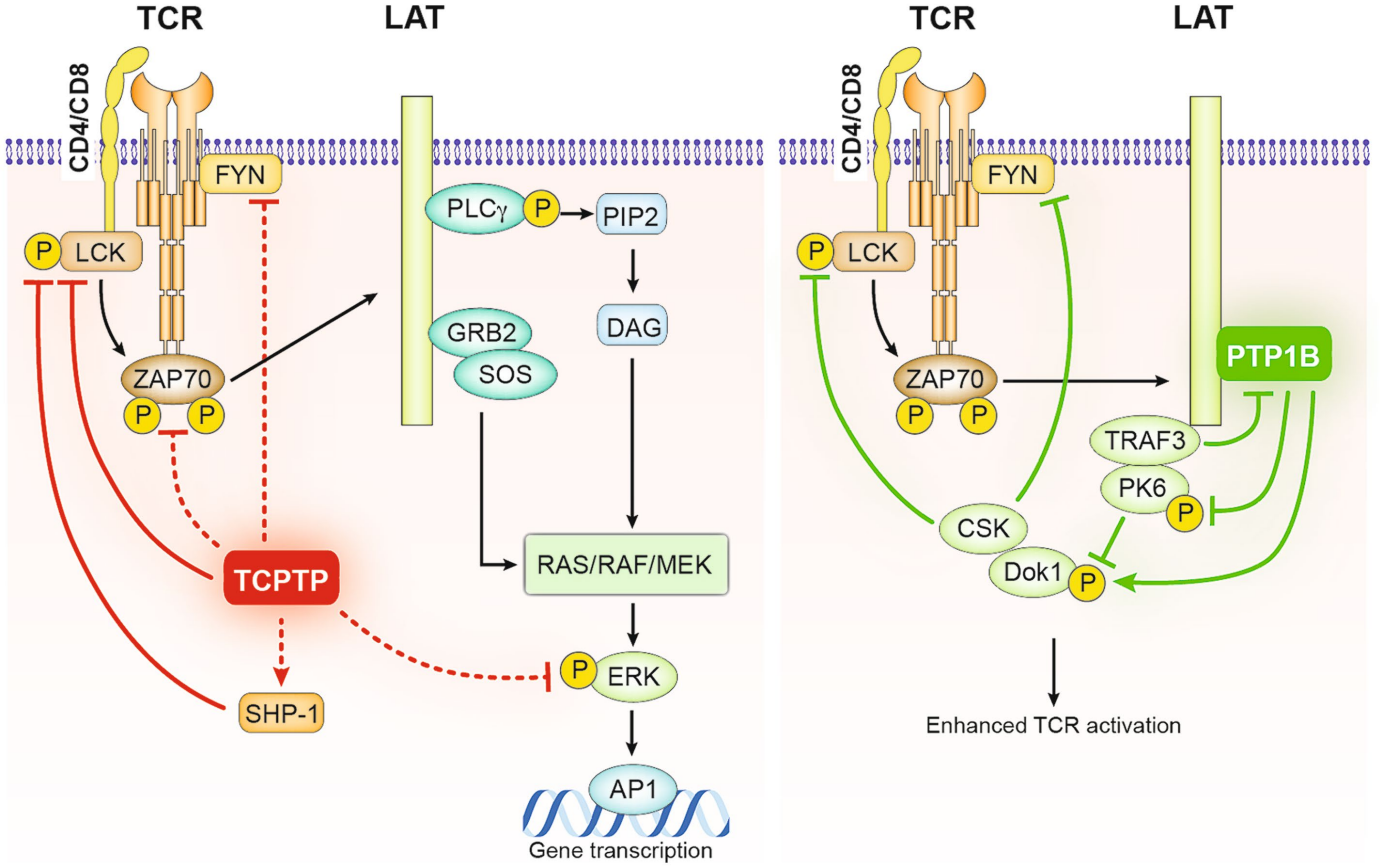
Negative regulation of TCR signaling in T cells by TCPTP and PTP1B. As illustrated on the left, Lck and Fyn serve as direct substrates for TCPTP in T cells. Following TCR engagement, TCPTP negatively regulates the initiation of TCR signaling by dephosphorylating Lck and Fyn and consequently decreasing the phosphorylation of downstream ZAP70 and ERK. It has been proposed that SHP-1, which also acts as a negative regulator of TCR-mediated activation of Lck and ZAP70, might be active downstream of TCPTP in these processes. Alternatively, PTP1B by associating with the adaptor LAT facilitates the inhibitory role of downstream adaptor DOK-1, − it does it by competing with TRAF3 on LAT binding and targeting the phosphorylation of PK6 and DOK-1 stabilizing it and allowing its recruitment of the inhibitory kinase CSK.

Evidence from mouse models with conditional deletion of TCPTP in T cells suggests that TCPTP negatively regulates reactivity to TCR ligation and alters the TCR threshold in naïve CD8 T cells by controlling the phosphorylation of the activating residues Y394 and Y418 of Lck and Fyn, respectively (31, 80). As a result, downstream phosphorylation of ERK1/2 (p-ERK1/2) is suppressed (31, 81) (Figure 2). Naïve CD8 T cells obtained from these mice were found to be hyperresponsive to *in vitro* TCR stimulation (31, 80). Similar findings applied to CD4 T cells. Deletion of TCPTP in human CD4 T cells using clustered regularly interspaced short palindromic repeats (CRISPR)/CRISPR-associated protein 9 (Cas9) led to increased intracellular calcium (Ca++) fluxes and expression of CD25, CD69, CD71, and PD1 in response to TCR engagement (87). Another piece of evidence supporting the role of TCPTP in controlling TCR signals is the direct correlation between the expression of CD5 and levels of TCPTP. This suggests that TCPTP may act as a counterbalancing mechanism in T cells expressing high-affinity TCRs (80). Similarly, higher levels of TCPTP expression are observed in memory CD8 cells, which respond more rapidly to TCR engagement (96). Nevertheless, in CD5 high naïve T cells, it has been established through the use of pharmacological inhibitors that CD45 rather than other PTPs, including TCPTP, is responsible for regulating TCR-induced signals (96).

In the case of PTP1B, recent evidence links it to a feedback loop that regulates TCR signaling. In platelets, PTP1B is colocalized with the adaptor protein LAT upon platelet activation through FcγRIIa stimulation (52) resulting in rapid dephosphorylation of LAT. In T cells, PTP1B appears to inhibit TCR signaling through a LAT dependent mechanism, involving other regulators of T cell activation, the adaptor protein tumor necrosis factor receptor-associated factor 3 (TRAF3), and the tyrosine kinase docking Protein 1 (DOK-1) (78) (Figure 2). TRAF3 is known to promote Lck activation and TCR signal initiation (78), whereas DOK-1 contributes to a LAT-dependent negative feedback loop that modulates early TCR signal (97). Notably, upon TCR engagement, the activation of LAT and the tyrosine phosphorylation of DOK adaptor proteins recruit other negative regulators of TCR such as the tyrosine phosphatase SHP-1 (94) and the inhibitory kinase C-terminal Src kinase CSK (98). In the absence of TRAF3, PTP1B was observed to impair TCR/CD28-mediated activation of SFKs by inhibiting DOK-1 degradation through the dephosphorylation of protein tyrosine kinase 6 (PTK6) (78). DOK-1 is also as a substrate for PTP1B (51), thus, PTP1B may reinforce the negative feedback loop in T cells by directly dephosphorylating DOK-1. TCPTP is also known to interact with TRAF3 at the cellular membrane, potentially modulating TCR signals through a similar mechanism (99). More recently, evidence from myeloid cells and 293 T overexpression experiments suggests that STAT3 dephosphorylation by PTP1B could be favored in the presence of TRAF3 in Granulocyte-macrophage colony-stimulating factor (GM-CSF)-mediated signaling (100) a mechanism that could potentially affect T cell signaling. Overall, these data support the idea that both TCPTP and PTP1B act as attenuators of TCR signaling, either by directly affecting TCR signaling components such as Lck or by acting on counterbalancing regulatory mechanisms.

## PTP1B and TCPTP in peripheral tolerance and T cell-mediated autoimmunity

The maintenance of peripheral tolerance is achieved through a range of T-cell intrinsic regulatory mechanisms including clonal deletion, anergy, and activation-induced apoptosis as well as extrinsic immune-suppression mechanisms by regulatory cells including regulatory T cells (Tregs). Any failure in these mechanisms may result in aberrant T cell responses to self-antigens and dysregulation of cytokine production leading to autoimmunity (101, 102). Several single-nucleotide polymorphisms (SNPs) in the TCPTP gene have been linked to disease susceptibility in type 1 diabetes (T1D), Crohn’s disease, Celiac disease, inflammatory bowel disease (IBD), and rheumatoid arthritis (103–108). The functional significance of TCPTP associated genetic variants in T cell-mediated immune tolerance and autoimmunity has been studied in both mice and humans.

In pre-diabetic NOD mice, TCPTP deficiency in T cells accelerated the onset of TD1, likely due to the infiltration of pathogenic cytotoxic CD8 T cells and activated insulin-specific IFN-producing CD4 T helper-1 (Th1) cells (82). Indeed, the enhanced Th1 polarization and aberrant activation of these cells are major contributors to organ-specific autoimmunity (101). The role of TCPTP in Th1/Th2 differentiation of CD4 T cell responses is further documented by other studies. *In vitro*, TCPTP deficiency in naïve CD4 T cells promote Th1 differentiation and results in augmented basal and IFN-γ-induced STAT1 phosphorylation (82, 83). When adoptively transferred into Recombination activating gene 1 and 2 (RAG1/2) deficient mice, these cells exhibit enhanced polarization into IFN-γ producing Th1 cells and IL-17 producing Th17 cells causing more aggressive dextran sulfate sodium (DSS)-induced colitis (83). These findings are further supported by the pronounced expression of Th1 and Th17 associated cytokines/transcription factors in the inflamed colons of Crohn’s disease patients carrying the risk allele rs1893217 (83).

**Figure 3:**
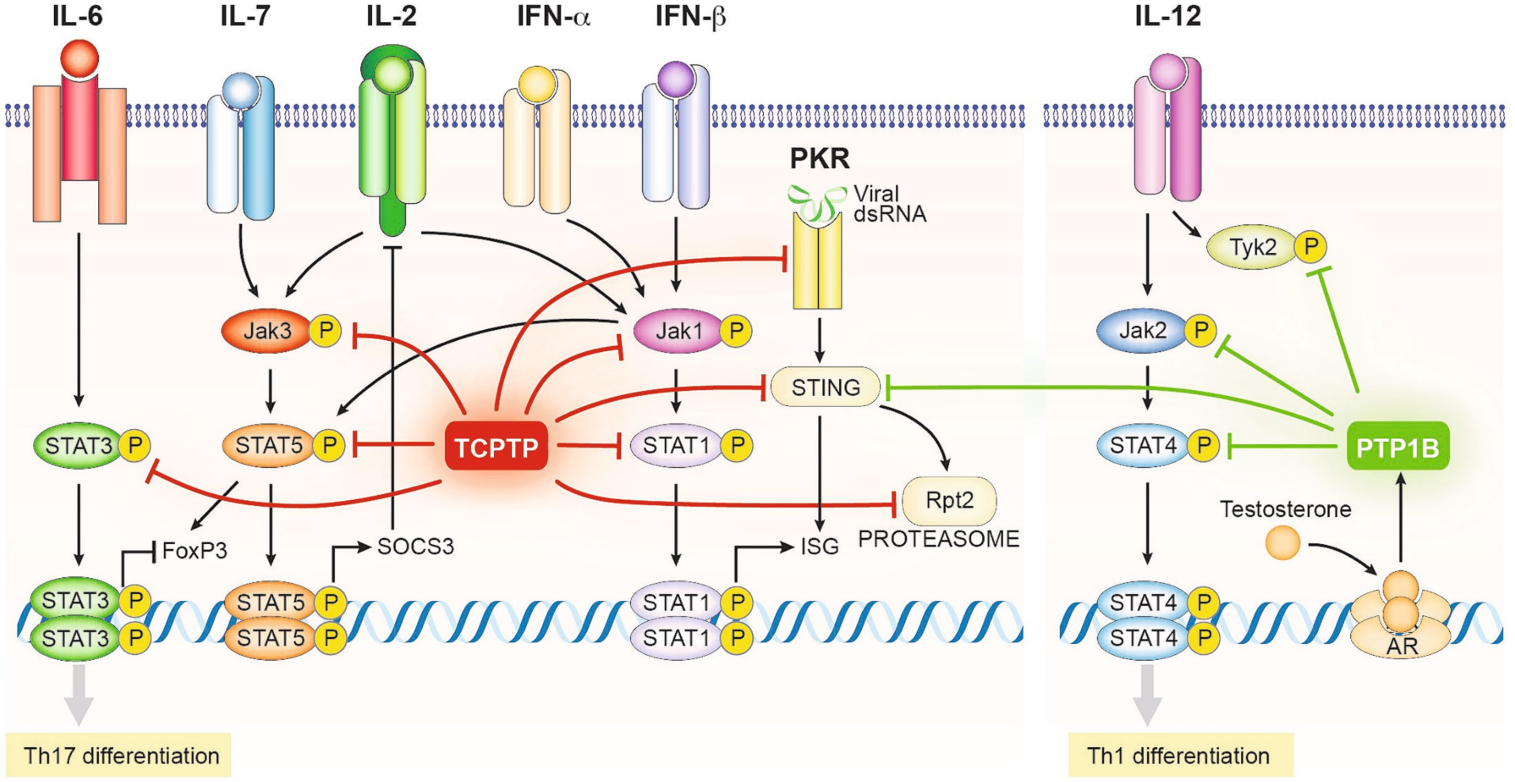
Schematic representation of the regulatory mechanisms driven by the enzymatic activity of TCPTP and PTP1B in T cells (Excluding those related to TCR signals depicted in Figure 2). TCPTP and PTP1B target at various levels the activating phosphotyrosine residues of several components of the JAK/STAT signaling network, including all JAK proteins and several STATs. Consequently, TCPTP and PTP1B restrict the onset of the Th1 and Th17 differentiation programs and the expression of interferon-stimulated genes (ISG). Alternatively, TCPTP and PTP1B can regulate other pathways with synergistic effects on its JAK/STAT roles, such as the viral transducer STING, the viral sensor PKR, and components of the proteasome. PTP1B expression is regulated by the androgen receptor limiting Th1 differentiation in males and explaining partially gender-related differences in immune reactivity.

Pathologic conversion of T regs into Th17 cells with partial loss of TCPTP was also observed in SKG mice, a genetic mouse model of spontaneous rheumatoid arthritis. Treg cells from these mice showed an increased pathogenic conversion into Th17 cells *in vitro* when stimulated with IL-6 (84). The IL-6 receptor (IL-6R) signals through Jak 1 and 2 kinases hence is susceptible to regulation by TCPTP. Downstream, IL-6R mainly induces the phosphorylation of STAT3 (Figure 3) which is known to participate in the dysregulation of forkhead box P3 (FoxP3) expression, the main driver of regulatory T cell (Treg) differentiation (84). Peripheral Tregs (pTregs) and Th17 cells share common precursors. In those, FoxP3 represses the expression of the Th17 inducing transcription factors, RAR-related orphan receptor gamma T (RORγt), and RORα (109, 110) favoring the Treg cell path. Conversely, as a consequence of reduced TCPTP activity observed in loss of function (LOF) mutations, increased phosphorylated STAT3 breaks FoxP3 repression, promoting Th17 differentiation. Interestingly, in this study no differences in TCR signaling were found in TCPTP haplodeficient mice in the C57Bl/6 and BALB/C backgrounds, strongly suggesting that effects on Treg differentiation are secondary to cytokine signal dysregulation. It is worth noting that, the conversion of existing Treg cells into Th17 cells upon TCPTP deficiency is observed in other models (111).

The impairment of IL-2/IL-2R signaling has also been linked to the breakdown of tolerance mechanisms contributing to the severity of autoimmune pathologies (112). IL-2 signals through JAK1 and JAK3 phosphorylating downstream different members of the STAT family, particularly STAT5 (13, 85, 113) (Figure 3). The LOF PTPN2 rs1893217 risk allele has been associated with reduced signaling through IL-2Rβ in response to IL-2 and IL-15, resulting in a decrease in pSTAT5 (88). Consistent with previous studies in mice (31, 114), the PTPN2rs1893217 risk allele was associated with decreased expression of CD25 by CD4 T cells and reduced pSTAT5 after IL-2 stimulation in carrier cells (88). While it may seem counterintuitive given the function of TCPTP, this aligns with other defects where suboptimal IL-2 signaling leads to the loss of tolerance in CD4 T cells by altering Treg differentiation (112), Nevertheless, consistent with these results, no disparity was observed in the numbers of circulating Tregs in human subjects carrying risk allele PTPN2 rs1893217. However, when these cells were activated in the presence of IL-2, impaired induction of the FoxP3 transcription factor and reduced activation in those Tregs were evident (86). A mechanistic explanation comes from novel evidence indicating that tonic signals induce the JAK/STAT regulator SOCS3 desensitizing TCPTP deficient T cells to IL-2 (87) (Figure 3).

Notably, in IL-2 induced T cells, the nuclear form of TCPTP physically interacts with its substrates JAK1 and JAK3. During precursor to mature Tregs transition in the thymus, TRAF3 was shown to act upstream of IL-2-induced STAT5 phosphorylation facilitating the recruitment of TCPTP to the IL-2 signaling complex. Hence, reducing the maturation of Treg precursors by limiting IL-2 signals (99). Results from different research groups suggested that TCPTP deficiency in T cells can directly affect the numbers of Tregs in the thymus and periphery depending on the mouse model (80, 83). In addition, TCPTP was induced by TCR signals in an *in vitro* model of induced Tregs (iTreg) cells. There, it was found to be involved in the Foxp3-depletion by controlling STAT5 phosphorylation (86). However, this effect was not observed in systemic thymic or peripheral Tregs limiting its physiological significance. In resume, LOF function mutations of TCPTP highlight the role of this phosphatase in T cell regulation, and by modeling these mutations with TCPTP haplodeficient mice, it was possible to define that the combined role of increased pSTAT3 induced by IL-6 and decreased pSTAT 5 after IL-2 stimulation participate in the dysregulation and pathogenic conversion of Tregs.

Decreased PTP1B activity has been also associated with decreased peripheral tolerance by inhibiting Th1 differentiation in a sex-dependent manner (115). PTP1B is known to regulate the phosphorylation and activity of JAK2 and non-receptor tyrosine-protein kinase 2 (TYK2), which transduce signals of the receptors for the Th1 inducing cytokines IL-12 and IL-23 (Figure 3). Testosterone and androgen analogs have been found to induce PTP1B expression, resulting in an androgen-based mechanism responsible for the inhibition of Th1 polarization. Additionally, increased PTP1B expression has been correlated with decreased IL-12 induced phosphorylation of STAT4 (77), thus suggesting a role for PTP1B in sex-related susceptibility to autoimmune diseases. Consistently, expression levels of PTP1B were increased in CD4 T cells from male patients undergoing androgen deprivation therapy (77).

## Intrinsic effects of TCPTP in CD8 T cell differentiation

When isolated and studied *in vitro*, CD8 T cells from the Lck-Cre mouse responded more vividly to standard CD3/CD28 antibody cross-linking *in vitro*. These cells proliferated faster and expressed higher levels of the activation markers CD69, CD25, and CD122, which are associated with increased phosphorylation of TCR components (116). Adoptive transfer experiments demonstrated that OT-1 naïve CD8 T deficient for TCPTP also exhibited enhanced proliferation in response to the cognate antigen OVA in syngeneic hosts (31). A role of TCPTP in reducing the TCR sensitivity threshold was confirmed by the elevated proliferative response of OT-1 TCPTP deficient CD8 T cells to a partial agonist OVA peptide. Furthermore, in the cross-presentation mouse model, rat insulin promoter (RIP)-mOVA, in which germline expression of OVA antigen induces anergy of OT-1 CD8 T cells to avoid self-reactivity (117), OT-1 TCPTP deficient CD8 T cells were able to escape tolerizing signals and induced disease, confirming an intrinsic defect of TCPTP deficient CD8 T cells (114).

To avoid developmental aberrations caused by early TCPTP deletion (31) and/or early expression of the CRE recombinase in thymocytes (118), LaFleur and colleagues developed a system using CRISPR/Cas9 recombination (32). In this model, adoptive transfer of mature naïve CD8 T cells lacking or not the expression of TCPTP from P14 mice (transgenic for a TCR recognizing the lymphocytic choriomeningitis virus (LCMV) gp33/Db) was used to treat mice challenged with the LCMV clone 13. Consistent with previous findings, TCPTP deleted CD8 T cells exhibited a significant increase in proliferation and expression of effector molecules, such as granzyme B at earlier time points. In addition to the increased initial proliferation, challenging TCPTP deficient OT-1 CD8 T cells with B16-OVA revealed enhanced effector qualities. Transcriptional analysis of these CD8 cells revealed an increased expression of IFN-α regulated genes in TCPTP deficient cells (32), thus confirming that mature CD8 T cells undergo transcriptional changes previously observed in models of T cell development (71). One of the best-described functions of TCPTP is the regulation of IFN-driven signals by controlling nuclear STAT1 phosphorylation, which has been observed in several other cell types (90, 119, 120). Therefore, it is possible that TCPTP deficiency leads to tonic STAT1 phosphorylation, resulting in the improved effector activity observed in TCPTP deficient CD8 T cells by simulating continuous type 1 IFN signals (Figure 3). These cells were characterized by the expression of T cell immunoglobulin and mucin domain-containing protein 3(Tim-3)+, a marker that characterizes a subpopulation of terminally differentiated CD8 T cells (32). Tim-3+ cells are described as highly cytotoxic but slowly proliferative cells (121), nevertheless, TCPTP deficient CD8 Tim-3+ cells proved to be highly proliferative. Type 1 IFN signals are required for the formation of Tim-3+ cells by opposing the T cell factor 1 (TCF1)- B-cell lymphoma 6 (BCL6) axis (122), strongly suggesting that dysregulation of these signals is the mechanism underlying the enhanced formation of these Tim3+ cells in TCPTP deficienct mice. Other models have also confirmed that dysregulation of alternative cytokine pathways collaborates with the onset of enhanced effector functions in TCPTP deficient CD8 T cells. An increased sensitivity to IL-2, IL-7, and IL-15 cytokines was found to be responsible for enhanced proliferation and permanence of terminally differentiated CD8 T cells identified by the alternative effector marker killer cell lectin-like receptor subfamily G member 1 (KLRG1) (81). These cells, as observed in the TCPTP deficient Tim-3+ cells, maintained expansion capabilities that the WT counterparts lacked (123). So far, different models show how the combined actions of TCPTP deletion in CD8 T cells result, as expected, in increased sensitivities to TCR and cytokine signals, notably, of those associated with the gamma chain receptor and type 1 interferons (depicted in Figure 3). As a consequence, CD8 T cells lacking TCPTP exert lower activating thresholds and maintain terminal effector properties without losing their proliferative capacity.

## TCPTP deficiency in CD8 T cell memory formation, bystander activation, and exhaustion

While in LCMV (32) and Listeria (81) infectious disease models the absence of TCPTP promoted a terminal effector phenotype, they did it at the expense of a reduced number of memory precursors. Memory formation in CD8 T cells can give rise to a non-circulating resident cell type observed in epithelial tissues, named T resident memory (Trm) cells. TCPTP deficiency in CD8 T cells prevented the formation of Trm cells in an HSV infection model (124). This restriction coincided with the accumulation of KLRG1+ effector CD8 cells, mirroring previous observations in LCMV and Listeria models. Clever manipulations with KLRG1 LOF mutants indicated that its inhibitory signals driven by its ITIM motif rather than deletion of TCPTP accounted for the block on Trm cell differentiation. Strengthening this finding, TCPTP deficient cells were able to produce KLRG1 negative effector cells, the precursors of Trm cells (125). Therefore, while deficiency of TCPTP promotes a terminal effector phenotype, it does not prevent the initiation of a memory program. It is then well understood that TCPTP plays a role in the regulation of TCR and proinflammatory cytokines in CD 8 T cells. Yet, a question arises on whether TCPTP deficiency imitates memory bystander activation. Bystander activation is an alternative form of activation that bypasses the TCR signals of memory CD8 T cells in response to type 1 and 2 interferons, IL-15 and IL-18 (126). This phenomenon is observed as a result of non-specific memory CD8 T cells exposure to highly inflammatory microenvironments and is well documented in human viral infections (127–129), and mouse models (130–132). Although the contributions to the success in the immune response remain controversial.

CD8 T cell exhaustion is a term coined to describe a hyporesponsive state that occurs when T cells are chronically stimulated, as is often seen in in infections such as HIV or the Hepatitis B virus, and cancer. This state is triggered by repeated engagement of activating signals (133). Exhausted T cells (Tex) have a unique transcriptional signature characterized by combined the expression of several inhibitory receptors as PD1, CTLA3, Tim-3, B-and T-lymphocyte attenuator (BTLA) and Lymphocyte Activation Gene 3 (LAG3) (5). As a general mechanism, these inhibitory receptors recruit tyrosine phosphatases as SHP1 and SHP2 to counteract the action of activating kinases on the TCR and co-stimulatory receptors (134). Since TCPTP and PTP1B are involved in downregulating signals from TCR and proinflammatory cytokines, it is reasonable to assume that they participate in the onset of exhaustion (5). However, recent data suggest that deletion of TCPTP increased the formation of terminally differentiated CD8 T cells instead of preventing exhaustion (32, 81). Notably, tumor-infiltrating TCPTP deficient OT-1 T cells have been shown to express reduced levels of exhaustion markers, PD-1 and LAG-3 during the late tumor development stages (89). Yet, it is to note that these TCPTP deficient populations exhibited better proliferative qualities than their WT counterparts, suggesting that TCPTP may have a potential role in the development of CD8 T cell exhaustion.

## Combine deletion of PTP1B and TCPTP

Homozygous germline deletion of both phosphatases proved to be embryonically lethal (120). However, the progeny of parents carrying heterozygous null alleles for both phosphatases produced expected Mendelian ratios for most of the possible genotypes. An exception, besides the double deficient mice, was found in those homozygous for TCPTP deletion and heterozygous for the PTP1B allele. Those mice were represented by half of the predicted proportion. Embryonic analysis showed that Mendelian ratios were conserved up to E9.5. After this stage, double-deficient embryos displayed growth retardation (120). Increased phosphorylation STAT1 characterized these embryos and was not observed in other genotypes while no differences in STAT3 or STAT5 phosphorylation were found. This evidence suggests that both PTPN1 and TCPTP participate in the control of STAT1 phosphorylation, either by sharing the same mechanism or by acting at different levels of the pathway. Regulation of STAT1 phosphorylation is key for the differentiation of T cells, IFN driven responses and Th1 skewing. Indeed, the inhibition of both of these enzymes in dendritic cells promoted the differentiation toward a DC1 type, with enhanced secretion of IL-12 and promoting increased T cell activation (1) suggesting that the combined action of both phosphatases regulates IFN signals independently of the cell type.

More recently, LaFleur and colleagues demonstrated that deficiency of both enzymes in immune cells was sufficient to induce lethal immunopathology through the use of A novel CRISPR-based technique, CHIME, which allowed the deletion of both genes in hematopoietic precursors (135). Lesions were characterized by changes in bone marrow cellularity, thymic atrophy, and decreased splenic white pulp areas, consistent with phenotypes seen in TCPTP KO mice and “TCPTP+/− PTP1B −/−“mice (35, 36, 120). They demonstrated that the lesion was dependent on the lymphoid lineage as it was absent in chimeras produced from RAG2-deficient mice (135). Sequential deletion (S-CHIME) or reduced chimerism revealed that in a mature immune system environment, combined deletion of TCPTP and PTP1B also induced pathologic inflammation which targeted the gastrointestinal and pancreas resulting in weight loss-related death.

## PTP1B and TCPTP as emerging targets for cancer immunotherapy

With a clear role in the regulation of proinflammatory TCR and cytokine signals, TCPTP and PTP1B have become of particular interest in areas where disruption tolerogenic signals is beneficial such as in cancer immunotherapy. Since the early observations made in the full-body KO mice of TCPTP (5, 35, 136) have led to the recognition of these phosphatases as intracellular checkpoints. More recently, there has been a growing understanding of their potential as targets in in immunotherapeutic approaches, such as enhancing of dendritic cell-based immunization or to improving T based cell therapies (1, 32, 89). Furthermore, the fact that the pathways regulated by PTP1B and TCPTP are independent of those involving CD28 regulation, such as PD1 or CTLA4, suggests that targeting these phosphatases pharmacologically could result in powerful combinatorial responses.

The effects of TCPTP deficiency in enhancing the tumor immunosurveillance of T cells have been tested in several mouse models of malignancy. One such model is the loss of heterozygosity (LOH) of the P53 tumor suppressor, which spontaneously develops different types of malignancies in almost half of the mice by 17 months of age (137). When these mice were bred with Lck-CRE conditional TCPTP deficient mice, tumorigenesis was completely absent at 1 year of age. In contrast, more than 50% of controls presented signs or findings of different active neoplastic growth (89). In the same publication, the authors reported the ability of mice with the same conditional deletion of TCPTP in T cells to clear solid tumors. In the orthotopic model of breast cancer AT-3 OVA, mice on the OT-1 background were challenged. As observed in the P53 LOF model, tumor growth was controlled in all animals with TCPTP-deficient T cells, while control mice exhibited tumor growth. Similarly, the growth of MC38 colon cancer tumors was completely inhibited in bone marrow chimeric mice with near to a 50% deletion of TCPTP in the hematopoietic compartment In these mice, the antitumoral activity was carried out by CD8 T cells, as tumor formation occurred when these cells were depleted. However, the observed role of CD4 T cells in tumor control cannot be ruled out given the capacity of TCPTP inhibition to dysregulate the formation and maintenance of Tregs, while promoting Th1 and Th17 differentiation of CD4 T cells, both known to be beneficial for tumor clearance (138, 139). Notwithstanding an impressive finding, the aging of T cell specific TCPTP deficient mice was affected by chronic inflammation and autoimmunity as previously reported (31, 89).

The concept of inhibiting TCPTP and PTP1B activity to potentiate adoptive transfer-based immunotherapies was first proposed in dendritic cells (1). In regards to T cells, it is well known that the cytotoxic activity of CD8 T cells is the goal of antitumoral responses and is the ground for tumor-infiltrating lymphocytes (TIL) therapy and chimeric antigen receptor-T CAR-T cell therapy (140). Based on the OT-1 model, the adoptive transfer of specific CD8 T cells deficient in TCPTP increased the survival and reduced the tumor burden of mice challenged with AT-3 orthotopic tumors (89). The authors further used a humanized model to test the ability of TCPTP deletion to modify the activity of human CAR-T cells in a setting closer to clinical conditions. A single dose of mouse HER-2 CAR-T cells, deficient or not in TCPTP, was used to treat orthotopic HER-2-E0771 breast cancer tumors. Notably, CAR-T cells expressing TCPTP were highly ineffective in controlling tumor growth and only marginally prolonged the lifespan of the challenged mice, up to a maximum of 25 days. In contrast, nearly 50% of female mice treated with TCPTP-deficient CAR-T cells survived beyond day 200 post-challenge. Additionally, despite TCPTP-deficient cells having a lower tendency to generate memory cells, mice that were rechallenged with HER-2-E0771 cells 70 days after treatment were still able to control tumor growth without any additional therapeutic intervention, demonstrating the presence of active memory.

Molecular evidence points to dysregulation of interferon signaling as one of the mechanisms driving increased responses in cells deficient in TCPTP. The phenotypic characteristics of T cells lacking TCPTP align with enhanced cytotoxic responses, which is consistent with observations made in other immune cell types such as bone marrow-derived dendritic cells (BMDCs) (1). BMDCs deficient in TCPTP exhibit differentiation features typically influenced by type 1 and 2 interferons, such as secretion of IL-12 and increased expression of Class I MHC molecules. It is to remark that in non-hematopoietic cells TCPTP is also a demonstrated regulator of interferon signals, and its deficiency equally promotes the expression of Class I MHC and decreases their resistance of tumor to anti-PD1 immunotherapy rendering them more susceptible to the action of CD8 T cells (90). Hence, inhibition of TCPTP and PTP1B is an immunotherapeutic approach that increases both the susceptibility of tumor cells to immune regulation and the activity of the anti-tumoral immune mechanism.

Besides their action on cytokine and TCR pathways, the modulatory role of TCPTP and PTP1B in other pathways can also contribute in immunotherapeutic settings. Such is the case for some stress response related sensors and transducers as stimulator of interferon genes (STING) (56) and protein kinase R (PKR) (141). In the case of STING, both TCPTP and PTP1B contribute to its degradation by the proteasome, limiting its activity (142) (Figure 3). STING is a transducer of cyclic GMP-AMP synthase (cGAS) signals, a sensor of double stranded cytoplasmic DNA found relevant in the activation of anti-viral and anti-tumoral responses (143). In the case of the viral RNA sensor PKR, TCPTP acts in a feedback loop that controls the presence of phosphorylated STAT1 in the nucleus after PKR activation (141). In both cases, absence of TCPTP and/or PTP1B can contribute to the amplification of Th1 type of signals acting in synergy with the enhanced sensitivity to type 1 IFNs.

The pathways regulated by TCPTP and PTP1B are independent of those already proven beneficial in cancer immunotherapy such as PD-1/PDL-1 or CTLA4, whose inhibitory signals are mediated by the phosphatases SHP-1 and SHP-2 (23). It allows the use of TCPTP and PTP1B inhibition as a combinatorial therapy. Indeed, deletion of TCPTP exerted synergistic effects of anti-PD-1 in the immune-refractory melanoma model B16 (32). In other demonstration of its potential use in combinatorial immunotherapy, TCPTP also potentiated the antitumoral properties of REGNASE-1 deficient CD8 T cells, a recently proposed intracellular checkpoint mediator (144).

## Pharmacological targeting of PTP1B and TCPTP

The identification of tyrosine phosphorylation and its dynamic components, kinases, and phosphatases, which make this protein modification reversible, have opened a remarkable number of biological and physiological systems to potential pharmacological modulation. Over the past three decades, more than 1,200 papers have been published in search of novel inhibitors for PTP1B, but only a few have demonstrated weak specificity for either TCPTP or PTP1B at best.

The difference and details of the pharmacological inhibition have been discussed in detail, for review see (145–147). On PTP1B, Tonks’ lab identified that the mechanism of inhibition of the MSI-1436 inhibitor was through binding on the C-terminus of the PTP which exerts allostery upon the binding site on the target facilitating proximity. Hence providing an excellent specificity of PTP1B over TCPTP. These conserved allosteric networks in PTP1B and TCPTP enables the modulation of both enzymes through dissimilar allosteric means (64). Moreover, by binding to specific sites, it is possible to enhance selectivity for one enzyme over the other. Allosteric inhibitors that target PTP1B demonstrate remarkable effectiveness and selectivity in comparison to other phosphatases (64). This highlights the potential of allosteric inhibition as a viable strategy in the development of selective inhibitors.

The desired PTP1B selectivity has been fueled by its promise as an important regulator of the insulin pathway, hence access to a well-established market of oral hypoglycemic compounds (146, 148). Despite the extensive literature, only a few molecules have made it to the preclinical and clinical trial stages, and none of them are currently available in the market. One of the main challenges, is the significant similarity between the catalytic domains of PTP1B and TCPTP, which hinders the development of substantial selectivity when targeting the catalytic site.

Is it crucial to achieve ‘inhibitor specificity’ between TCPTP and PTP1B? In the late 90s, Merck-Frosst pursued PTP1B inhibition by simultaneously binding the catalytic pocket and the adjacent pseudo-phospho-tyrosine binding pocket, which is only present in PTP1B or TCPTP. In this, they were successful in developing compounds that had some preferences for PTP1B. These bifluoride-containing chemical entities still recognized TCPTP but remarkably did not significantly inhibit any of the other members of the PTP family (149). The complementation of these two enzymes in the production of IL-1 provides means to shortcut the need for specific inhibitors for PTP1B or TCPTP. This would be especially useful in the activation of immune cells as described above.

Calico Life Science proposed a novel series of compounds with similar inhibitory specificities, which are currently being tested in clinical trials by AbbVie. These compounds, including Compound 182 and ABBV-484, showed remarkable results in preclinical studies (3, 4). In these studies, Compound 182 and ABBV-484 were able to promote diverse tumor eradication either alone or in combination with anti-PD1, through diverse immune mechanisms involving T cells, NK cells, and tumor intrinsic sensitization to interferon induced dead. Treatment with compound 182 did not induce immunopathology at the acute level revealing a potential wide therapeutic window before double inhibition of PTPN1 and TCPTP could become deleterious, however, this was assessed only in the acute phase. A detailed account of compounds inhibiting PTP1B and/or TCPTP with demonstrated preclinical evidence or advanced to clinical trials is listed in Table 2, however, to the date of writing (March 16, 2024) no results have been published from oncology related studies.

**Table 2:** TCPTP and PTP1B inhibitors advanced in cancer therapeutics.

Inhibitor	IC_50_(μM)		Company/ Developer	Preclinical evidence (*in vivo*)*	Clinical trials	Mechanism of action
	**PTP1B**	**TCPTP**				
Trodusquemide (MSI-1436)	1	224	DepYmed	BT474 (65)	NCT00509132^†§^ NCT00606112^†§^ NCT00806338^†§^ NCT02524951^#^**	Allosteric, Non-competitive
1B/TC inhibitor	–	–	Merck	E.G7 (*Ex vivo* treated OVA pulsed MoDCs) (1)	–	Competitive, Reversible
KQ-791	0.175	0.163	Kaneq/Kanyr	B16F10 (150), EMT-6 (151)	NCT02370043^†¶^ NCT02445911^†¶^	Competitive, Reversible
ABBV-CLS-484	0.0025	0.0018	AbbVie/ Calico	4 T1, EMT-6, B16F10, KPC (4)	NCT04777994^#‡^	Competitive, Reversible
ABBV-CLS-579	–	–	AbbVie/ Calico	–	NCT04417465^#‡^	–
Compound 182	0.00063	0.00058	AbbVie/ Calico	AT3 and AT3-OVA, MC38 (3)	–	Competitive, Reversible
PTPN2 Inhibitor 9	–	–	UCSD	B16F10 (152)	–	–

*BT474: Breast ductal carcinoma (human xenograft, systemic treatment); E.G7: OVA expressing Lymphoma (based on syngeneic EL4 cells); B16F10: Murine melanoma (Syngeneic); EMT-6: Murine mammary carcinoma (Syngeneic); 4 T1 Murine metastatic mammary carcinoma (Syngeneic); KPC: Pancreatic ductal carcinoma (Syngeneic); AT3 Murine mammary carcinoma (Syngeneic); AT3-OVA: OVA expressing AT3 cells; MC38: Colon adenocarcinoma (Syngeneic). ^†^For use as a medication for type 1 and/or type 2 diabetes. ^#^For use in oncology. ^‡^Currently ongoing, no results published. ^§^Completed, no results. ^¶^Completed, has results. **Terminated.

The use of these inhibitors holds a great promise in immunotherapy; however, it is still necessary to validate whether employing them as systemic treatment could cause overwhelming inflammatory response in patients. Even more worrisome would be the potential to further activate JAK/STAT signaling in transformed cells that depend on this pathway to expand. The inhibition of both, PTP1B and TCPTP, in B cell lymphoma, T cell acute lymphoblastic leukemia, and other potentially susceptible tumor types, may also negatively influence the disease outcome (24). Likely, systemic applications of those inhibitors will likely need to be targeted toward tumors with known genetic alterations that are independent of JAK/STAT signaling.

Alternatively, data from Penafuerte et al. (1), suggests that autologous and tolerogenic immune cells could be activated by *ex-vivo* treatment before the reintroduction of those cells in patients. The great advantage of a cell therapy approach is that treating with an inhibitor of PTP1B and TCPTP leads to a temporal change in the transcriptome of these immune cells. Hence, washing the compound away, before autologous cell transfer could provide significant benefits from the immune activation without the collateral risks of activating tumor cells, and potential hyper-inflammatory responses (1). Interestingly, hastening the use of such *ex-vivo* inhibitors as ancillary reagents is more easily acceptable by clinical trial regulatory agencies.

## Future perspectives

Herein, we have summarized the roles of TCPTP and PTP1B in the regulation of T cell responses. Their major role in the development, activation, and differentiation of T cells provides exciting possibilities for immunotherapeutic approaches. Their T-cell intrinsic properties are compounded by their positive effects on activators of T cells such as DCs and macrophages, as well as their capacity to sensitize tumoral targets to the activity of these cells. Moreover, studies in double mutants strongly suggest that concomitant inhibition of both phosphatases potentiates the effects already observed for TCPTP softening the requirement for small molecule inhibitor specificity. Hence, opening the door for pharmacological intervention and combination therapies using first-in-class immune activators through inhibition of both protein tyrosine phosphatases.

The encouraging preclinical results demonstrated by double inhibitors of PTP1B and TCPTP also provide reassurance for the phosphatase field as a whole, helping to define the pharmacological potential of these enzymes. Recent developments in allosteric targeting of SHP2 are also promising for oncological and autoimmune therapeutics proposing alternative strategies for more selectivity (153, 154).

## Author contributions

LP-Q: Conceptualization, Data curation, Formal analysis, Investigation, Writing – original draft, Writing – review & editing. BA: Conceptualization, Data curation, Formal analysis, Investigation, Writing – original draft, Writing – review & editing. MT: Conceptualization, Funding acquisition, Writing – original draft, Writing – review & editing.
